# Protect Your Sleep When Work is Calling: How Work-Related Smartphone Use During Non-Work Time and Sleep Quality Impact Next-Day Self-Control Processes at Work

**DOI:** 10.3390/ijerph15081757

**Published:** 2018-08-15

**Authors:** Lilian Gombert, Anne-Kathrin Konze, Wladislaw Rivkin, Klaus-Helmut Schmidt

**Affiliations:** 1 Leibniz Research Centre for Working Environment and Human Factors at the Technical University Dortmund, 44139 Dortmund, Germany; konze@ifado.de (A.-K.K.); w.rivkin@aston.ac.uk (W.R.) schmidtkh@ifado.de (K.-H.S.); 2 Work and Organizational Psychology Department, Aston University, Birmingham B4 7ET, UK

**Keywords:** daily diary study, ego depletion, self-control, sleep, spillover, three-way interaction, work-related smartphone use

## Abstract

In view of the rapid development of information and communication technologies, the present study sheds light on how work-related smartphone use during non-work time affects employees’ subsequent working day. Specifically, we examine work-related smartphone use and sleep quality as moderators of next-day self-control processes at work. Theorizing that work-related smartphone use and self-control demands deplete a common limited regulatory resource, we suggest a strengthening two-way interaction between work-related smartphone use during non-work time and next-day self-control demands at work in predicting employees’ ego depletion at work. Moreover, in a three-way interaction, we analyze whether this interaction depends on employees’ sleep quality, assuming that when intensive work-related smartphone use is followed by high-quality sleep, the taxed regulatory resource can replenish overnight. Results from our diary study covering 10 working days (*n* = 63) indicate that after evenings with high work-related smartphone use, employees experience disproportionate levels of ego depletion when dealing with self-control demands at work. Sleep quality, however, attenuates this interaction. In cases of high sleep quality, next-day self-control processes at work are no longer affected by work-related smartphone use. Based on these findings, we discuss implications for employees and employers regarding work-related smartphone use and the relevance of sleep in replenishing drained resources.

## 1. Introduction

Today’s working life is characterized by global competition, increasing service orientation, and the need to adjust quickly to changing markets [1]. These demands cannot be met by automated and rigid patterns of behavior. Rather, they call for considerable self-control [2]. Self-control refers to the ability to regulate one’s thoughts, emotions, and behaviors in order to align them with goals, rules, or other standards. Despite its benefits for effective functioning at the workplace, self-control is not without drawbacks. According to the Limited Strength Model of Self-Control [3], all acts of self-control draw on a common regulatory resource, which is limited and gets depleted by use. The depletion of this resource, called *ego depletion*, can temporarily impede subsequent self-control efforts, and is characterized by feelings of low willpower and cognitive exhaustion [4]. Once depleted, individuals need to recover in order to regain full self-control strength. If there is no opportunity for recovery, long-term consequences, such as chronic impairments in psychological well-being, may evolve.

This notion points towards demands on self-control as a source of stress at work. Accordingly, research on occupational health has provided convincing evidence that the demands to control impulses, to resist distractions, and to overcome inner resistances at work (summarized as self-control demands) relate to short-term (e.g., ego depletion [5]) and long-term (e.g., burnout, depressive symptoms, and absenteeism [2]) indicators of impaired well-being.

Yet, the rapid development of information and communication technologies in recent years has given rise to further job-related demands that necessitate self-control efforts, such as work-related smartphone use after official working hours. Smartphones enable employees to perform their work outside the confines of an office, and have thus become a prevalent technology to stay connected to customers, co-workers, and supervisors even during non-work time [6]. As such, this new technology causes employees to balance an extended availability for their job with private life, and to flexibly adapt their behavior according to either work or family roles (and the associated expectations). These characteristics suggest that work-related smartphone use during non-work time requires executive self-control processes, and in that way depletes limited regulatory resources.

**Figure 1:**
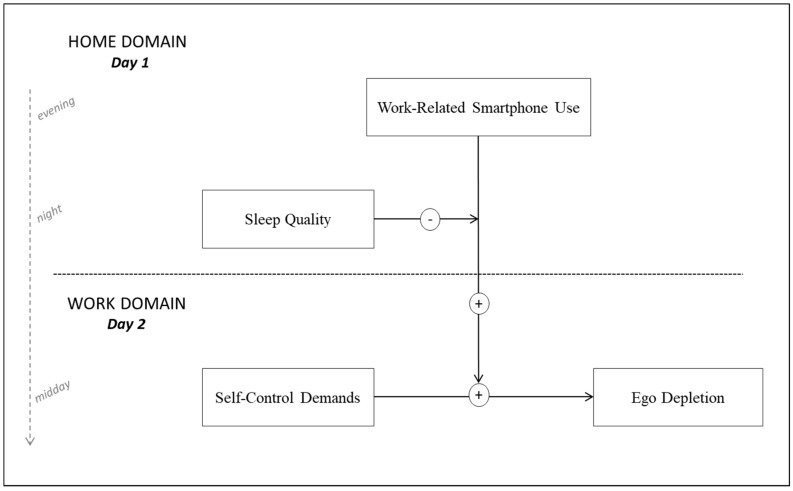
Research Model.

We aim to advance this proposition in our research by shedding light on the day-to-day consequences of work-related smartphone use from a self-control perspective. More precisely, using a daily diary research design, we examine how work-related smartphone use during non-work time (i.e., in the evening at home) affects self-control processes during the following working day. Figure 1 depicts the hypothesized study model. Integrating previous findings (a) that the use of limited resources in one domain (e.g., at home) reduces the availability of these resources in the other domain (e.g., at work, referred to as spillover [7]), and (b) that multiple demands on self-control can overtax the limited resource and thereby reinforce each other’s impact on resulting ego depletion [8,9], we propose that work-related smartphone use during non-work time interacts with next-day self-control demands at work in predicting employees’ experience of ego depletion at work. In other words, we assume that work-related smartphone use makes employees more vulnerable to the depleting effects of self-control demands at work. Moreover, given that time for recovery (i.e., appropriate rest [10]) can restore depleted regulatory resources, we introduce sleep quality as a driver of resource replenishment that may moderate the proposed interaction between work-related smartphone use and self-control demands. We argue that if employees experience high sleep quality after having used their smartphones for work intensively, the regulatory resource can recover overnight. As a consequence, self-control processes at work on the next day should no longer be affected.

The present study may offer several contributions to scholarly knowledge. First, whereas previous research has mainly focused on consequences of work-related smartphone use occurring in the home domain, such as depletion on the same evening or the next morning [11,12,13], we provide insight into how smartphone use during non-work time can affect the subsequent working day. In that way, our study may contribute to a holistic evaluation of the adverse effects of work-related smartphone use and broaden knowledge on the interconnectedness between home and work domains. Second, we intend to provide additional support for the notion that work-related smartphone use drains regulatory resources by investigating potential interaction effects with self-control demands in the prediction of ego depletion. We believe that understanding the process, which underlies the adverse effects of work-related smartphone use, is an important step towards ensuring employees’ well-being in modern work environments. Third, whereas previous studies revealed interaction effects of chronic demands on self-control in the prediction of long-term depletion [8,9], the present research examines whether such interactions can also manifest on a daily basis (i.e., in predicting daily levels of ego depletion). Using a daily diary approach allows for identifying more fine-grained differences in resource allocation that occur on the within-level, and could further contribute to a better understanding of the causality of these effects [14]. Finally, we address the lack of knowledge regarding how employees can protect themselves on days with intensive work-related smartphone use. Demonstrating that employees’ sleep quality mitigates regulatory resource depletion effects (and in that way fosters subsequent self-control processes at work) might hold valuable implications for both individuals and managerial practice.

### 1.1. Self-Control Demands: A Source of Stress at Work

During the last several years, self-control demands at work have received increasing attention in the literature on occupational health. Several studies have demonstrated that self-control demands (that is, controlling impulses, resisting distractions, and overcoming inner resistances) constitute an influential stressor at work [15]. Controlling impulses refers to the demand to inhibit spontaneous, impulsive response tendencies and associated affective states, which manifest, for example, in injudicious expressions towards other individuals. Resisting distractions involves the requirement to ignore interruptions evoked by task-irrelevant stimuli, which would interfere with successful task accomplishment (e.g., social media or private phone calls). Overcoming inner resistances relates to the requirement to overcome motivational blockades, for example in cases of unattractive tasks [15]. There is strong evidence from cross-sectional and longitudinal studies that self-control demands at work predict long-term indicators of impaired well-being (e.g., burnout, depressive symptoms) and reduced productivity (e.g., absenteeism [2]).

While most of the previous studies have focused on self-control demands as stable (i.e., chronic) characteristics of a given job, more recent research argues that self-control demands can also vary between working days [5,16]. More precisely, on days with intensive customer contact, frequent interruptions (e.g., by other individuals or high levels of noise at the workplace), or unattractive duties at work, required self-control efforts may be higher than on days with hardly any contact to other individuals. To capture these daily fluctuations, scholars have used daily diary designs in which self-control demands are assessed directly at work (e.g., in the afternoon) over several working days. The results indicate that self-control demands on a specific day can immediately translate to short-term indicators of impaired well-being (e.g., lower subjective vitality, higher levels of ego depletion) on this day [5,17].

The adverse effects of self-control demands can be explained by the Limited Strength Model of Self-Control [3]. This model suggests that each person’s capacity for self-control appears to be a limited, renewable resource. Various acts of self-control rely on that limited resource and deplete it with use, leaving less resource capacity available for subsequent self-control efforts. Self-control is thus costly in the short run and subject to fluctuations in capacity [18]. If prolonged self-control efforts prevent resource-replenishment, there is a risk of long-term consequences, such as burnout [2]. In sum, the notion that both chronic and day-specific self-control demands at work deplete regulatory resources and impact employees’ well-being is well-established in current literature.

### 1.2. Joint Effects of Work-Related Smartphone Use at Home and Self-Control Demands at Work on Ego Depletion

New communication technologies, such as smartphones, continually blur the traditional spatial and temporal boundaries of work. They allow employees to manage their calendar, speak to colleagues or clients on the phone, and to receive and answer e-mails wherever they are. As a consequence, many employees use their smartphone to stay connected to their job after the official end of working hours [19]. Recent studies have outlined that, despite some benefits (e.g., a higher productivity [20]), work-related smartphone use after official working hours holds risks for health and well-being. More precisely, employees who intensively use their smartphones for work during non-work time report reduced sleep duration [6] as well as difficulties to detach from work and to recover from work-related stress [11]. In addition, empirical evidence suggests that work-related smartphone use is positively related to work-family conflict [13], negative affect [21], and burnout [19]. Many of these studies have used diary designs to outline the varying intensity of smartphone use between different days (e.g., due to high work-load or upcoming deadlines) and its relation to daily indicators of impaired well-being.

Scholars argue that the adverse effects of work-related smartphone use during non-work time can be traced back to the consumption of self-control resources. More precisely, because employers have raised their expectations regarding employees’ availability, individuals may generate compulsive routines of checking for work-related phone calls and messages during off-job time in order to align with their work tasks and requirements. In that way, work-related smartphone use may require employees to maintain attention during leisure time although they might already feel exhausted (i.e., monitor and control themselves). As another example, work-related smartphone use can be assumed to introduce competing goals between work and private life. Individuals try to achieve organizational goals as they expect to receive positive outcomes in return, but at the same time might suppress personal goals and the own (or the partner’s/family’s) wish for private time without work. Managing such conflicts (e.g., deciding whether to check incoming messages when the smartphone rings or lights up during non-work time), however, has been demonstrated to involve acts of self-control [8,22]. Taken together, work-related smartphone use is highly likely to draw on and deplete limited regulatory resources similar to other demands on self-control.

Theoretical notions [3] as well as empirical findings [8,9] indicate that coping with multiple demands on self-control in a short period of time causes higher levels of depletion than accounted for by the additive effects of each demand, because the limited regulatory resource becomes overtaxed [9]. Accordingly, Schmidt [8] found that the adverse effects of self-control demands at work on impaired well-being are amplified when employees additionally experience high goal incongruence at work (i.e., a perceived mismatch between personal and organizational goals). Diestel and Schmidt [9], moreover, demonstrated strengthening interaction effects between self-control demands and emotional dissonance (i.e., suppressing genuine feelings in order to express organizationally desired emotions [23]) in predicting burnout, depressive symptoms, and absence behavior. Based on longitudinal or cross-sectional data, these studies focused on between-person effects, which indicate that adverse effects of chronic demands on self-control at work can cumulate to disproportionate levels of impaired well-being over time. However, little attention has been paid to whether these strengthening interaction effects also arise on a daily basis, that is, whether daily demands on self-control cumulate to higher levels of daily depletion.

To address this issue, and to extend knowledge on how work-related smartphone use affects employees’ self-control processes at work, we examine joint (interaction) effects of work-related smartphone use and self-control demands in the prediction of ego depletion. Supposing that both demands deplete a common limited regulatory resource, we propose that work-related smartphone use at home (Day 1, cf. to Figure 1) strengthens the adverse effects of next-day self-control demands at work (Day 2) on next-day ego depletion at work (Day 2).

According to Edwards and Rothbard [7], drawing on a limited resource in one life domain reduces the availability of this resource in the other domain. This notion implies that decrements in the regulatory resource may endure for some time and transcend the boundaries between home and work. We thus argue that on days when an employee intensively uses his or her smartphone for work-related purposes in the evening at home, he or she expends some of the limited regulatory resource. When this employee subsequently is confronted with high self-control demands at work on the following day (e.g., due to a monotonous task or a demanding client), the already decreased regulatory resource is taxed again. Consequently, because of lower resource availability, we assume that coping with self-control demands will be more straining when preceding work-related smartphone use has been high. In sum, coping with both daily work-related smartphone use during non-work time and self-control demands at work the next day should result in higher levels of ego depletion than accounted for by the additive effects of both demands.

**Hypothesis** **1.** Work-related smartphone use during non-work time moderates the day-specific relation between next-day self-control demands at work and next-day ego depletion at work. The relation is amplified when work-related smartphone use is high.

### 1.3. The Protective Function of Sleep Quality: Recovering the Limited Self-Control Resource

Given the costs of self-control efforts for employees’ well-being, research on occupational health has broached the issue of how depleted regulatory resources can be recovered. Since recovery processes naturally occur during rest [10,24], several scholars introduced sleep quality as one essential driver of day-to-day resource restoration [3,24,25,26]. High-quality sleep (i.e., for instance, easily falling asleep, staying asleep, a small number of awakenings during the night [24]) allows for full rewinding from work-related effort and a reduction of responsiveness, so that affective and energetic resources can return to pre-stressor levels [27]. Moreover, neurophysiological studies suggest that high sleep quality stabilizes the cerebral metabolic rate and ensures adequate resource or energetic supply of the prefrontal cortex, whose structures are essential for self-control functioning [28,29].

In line with this proposition, high sleep quality has been demonstrated to buffer the adverse consequences resulting from demands on self-control. For instance, Diestel and colleagues [30] found that sleep quality diminishes the impact of emotional dissonance on daily psychological well-being (ego depletion, need for recovery, and work engagement). Liu and colleagues [31] further revealed that the association between customer mistreatment and unhealthy eating (as an indicator of self-control failure) becomes weaker as a function of sleep quality.

On the basis of these findings, we believe that sleep quality may have the potential to offset the adverse consequences of work-related smartphone use. We thus investigate daily sleep quality as a buffer of the interaction effects of work-related smartphone use and self-control demands on ego depletion. Considering high-quality sleep as a daily resource-restorative process, we propose that work-related smartphone use during non-work time (Day 1, cf. to Figure 1) only amplifies the next-day relation between self-control demands and ego depletion at work (Day 2) when sleep quality (in the night between Day 1 and Day 2) is low. We argue that when an evening with high work-related smartphone use at home is followed by a night with low sleep quality, recovery processes are impeded so that employees return to work with insufficiently restored regulatory resources [32,33]. As a result, self-control processes at work are affected in the way that employees are more vulnerable to the depleting effects of self-control demands due to their drained resources, manifesting in disproportionate levels of ego depletion (as described in Hypothesis 1). In contrast, when sleep quality during that night is high, employees can replenish their regulatory resources overnight so that the full strength is available on the following working day. As a consequence, self-control processes at work (i.e., handling self-control demands) should no longer be influenced by preceding levels of work-related smartphone use. We thus propose that the interaction between work-related smartphone use during non-work time and self-control demands at work on employees’ ego depletion is attenuated as a function of sleep quality.

**Hypothesis** **2.** Sleep quality moderates the day-specific interaction between work-related smartphone use during non-work time and next-day self-control demands at work on ego depletion at work. In cases of low sleep quality, work-related smartphone use moderates the positive relation between self-control demands and ego depletion, whereas in cases of high sleep quality, work-related smartphone use and self-control demands do not interact in predicting ego depletion.

## 2. Materials and Methods

### 2.1. Participants and Procedure

We conducted a daily diary study in Germany to test our hypotheses. Participants were recruited through personal contacts, announcements on the authors’ institute homepage, and social networks. All participants received a compensation of 50 Euro. The final sample includes 63 employees, among which 57% (*n* = 36) were female. The mean age was 39.5 years (SD = 13.46) with a range from 20 to 64 years. Most participants worked in the service sector and had regular contact with customers, clients, patients, or other individuals. Occupations ranged from, for example, salespersons to consultants. Of the participants, 16% worked part-time (part-time employees in this study worked less days per week, but had full working days). Shift workers were not included in the study.

We collected our data by means of online surveys, which could be completed using smartphones, tablets, or computers. In advance of the day-specific measurements, participants answered a demographic questionnaire. They then received emails two times per day (in the morning at home, in the afternoon at work) over 10 consecutive working days including instructions and links to the day-specific questionnaires. The morning survey measured work-related smartphone use during off-job time in the previous evening and sleep quality. In the afternoon, participants were invited to rate self-control demands referred to the “last few hours of work” and current levels of ego depletion. On weekends or public holidays, the diary study was suspended and continued on the next regular working day. Overall, the response rate to our daily questionnaires was 97%, resulting in 603 out of 630 possible day-specific measurement points. The study was conducted in accordance with the Helsinki Declaration of 2008.

### 2.2. Measures and Control Variables

Age and gender were assessed and included in the analyses, as past research has indicated that demographics may be associated with day-level well-being [10].

*Work-related smartphone use during non-work time* was measured with the smartphone use scale developed by Derks and Bakker [19] adjusted for daily measurement. The scale consists of four items, which are scored on a five-point rating format (1 = *totally disagree*; 5 = *totally agree*) and include an explicit reference to work-related smartphone activities. Participants were instructed to answer the questions in the morning, referring to the previous evening (e.g., “Yesterday, I used my smartphone intensively during after work hours for work-related purposes”.). This approach follows previous research on work-related smartphone use [6].

*Sleep Quality* was assessed with the following item adapted from Buysse and colleagues [34]: “How do you evaluate this night’s sleep?”. This item has been used successfully in similar diary studies [32,35]. Participants rated their overall sleep quality on a four-point rating format (1 = *very poor*; 4 = *excellent*).

*Self-control demands* were measured with 15 items developed by Schmidt and Neubach [36]. On a five-point rating format (1 = *not at all*; 5 = *a great deal*), participants evaluated the requirements to inhibit impulses, resist distractions, and overcome inner blockades within the last hours of work. Exemplary items are “In the last hours, my job required me not to lose my temper” for impulse control, “In the last hours, my work required me to resist distractions” for resisting distractions, and “In the last hours, some of my tasks were such that I really needed to force myself to get them done” for overcoming inner resistances. The scale score was computed as the average of the single-item scores [37].

*Ego Depletion* was assessed using five items related to participants’ *current* experiences with resource depletion (e.g., “At the moment, I feel less able to focus on anything.”). Bertrams and colleaguesr [4] developed this scale in order to assess the psychological state of ego depletion proposed by Muraven and Baumeister [3]. All items are scored using a four-point rating format (1 = *not at all*; 4 = *a great deal*).

### 2.3. Construct Validity

Multilevel confirmatory factor analyses were conducted to test the psychometrical distinctiveness of our variables. We tested a four-factor model, which specified the three multi-item measures as latent constructs (self-control demands, work-related smartphone use, and ego depletion) and the single-item measure (sleep quality) as an observed variable. Therefore, we created parcels of the latent constructs by aggregating the item indicators (self-control demands (three parcels), work-related smartphone use (two parcels), and ego depletion (two parcels)). This practice offers a number of advantages, such as a reduced number of parameters, more normally distributed and reliable measures, and more efficient parameter estimates [38,39]. Fit indices indicated a good fit for the four-factor model (χ^2^ (44) = 215.72, *p* < 0.01; root mean square error of approximation (RMSEA) = 0.091; comparative fit index (CFI) = 0.940; standardized root mean square residual within-person/between-person (SRMRw/SRMRb) = 0.055/0.058).

### 2.4. Strategy of Analyses

We used multilevel modeling because our day-level data (Level 1) were nested within the person-level data (Level 2), and this procedure takes the interdependence of both levels into account [40]. All parameter specifications and estimates were conducted with the MLwiN software [41]. We specified several models to test our hypotheses. The null model only included the intercept. In Model 1, we added the person-level control variables age and gender. Model 2 additionally included daily work-related smartphone use, sleep quality, and self-control demands. In Model 3, we tested the proposed interaction between work-related smartphone use and self-control demands by including a cross-product term of both predictors. Finally, Model 4 included the remaining two-way interaction terms (self-control demands × sleep quality, work-related smartphone use × sleep quality) as well as the three-way interaction (self-control demands × work-related smartphone use × sleep quality). Since we were solely interested in day-specific effects, we centered self-control demands, work-related smartphone use, and sleep quality around the person mean (group-mean centering [42]) in order to remove any between-person variance. Age and gender as person-level variables were centered around the grand mean to reduce the risk of multicollinearity [42].

## 3. Results

Table 1 reports descriptive statistics, internal consistencies (Cronbach’s alpha), and correlations among the study variables. All measures revealed satisfactory consistencies. In order to examine the proportion of variance that is attributed to the different levels of analysis, we calculated the intra-class correlation (ICC) for the day-level variables. The high proportions of within-person variation (34% for work-related smartphone use, 73% for sleep quality, 27% for self-control demands, and 49% for ego depletion) justify the application of multilevel modelling. As depicted in Table 2 (Model 2), all study variables are directly related to ego depletion at work (work-related smartphone use: γ = 0.09, *p* < 0.05; sleep quality: γ = −0.12, *p* < 0.01; self-control demands: γ = 0.29, *p* < 0.01).

### 3.1. Testing the Two-Way Interaction between Work-Related Smartphone Use and Self-Control Demands

Hypothesis 1 predicted a day-specific interaction between work-related smartphone use and next-day self-control demands at work on next-day ego depletion at work. In support of this hypothesis, the proposed interaction is significant (γ = 0.32, *p* < 0.01; cf. Model 3 in Table 2). Moreover, a log-likelihood ratio test additionally indicates that adding the interaction term improved data fit (Δ − 2*log(df) = 6.708(1); *p* < 0.05) as compared to Model 2.

**Figure 2:**
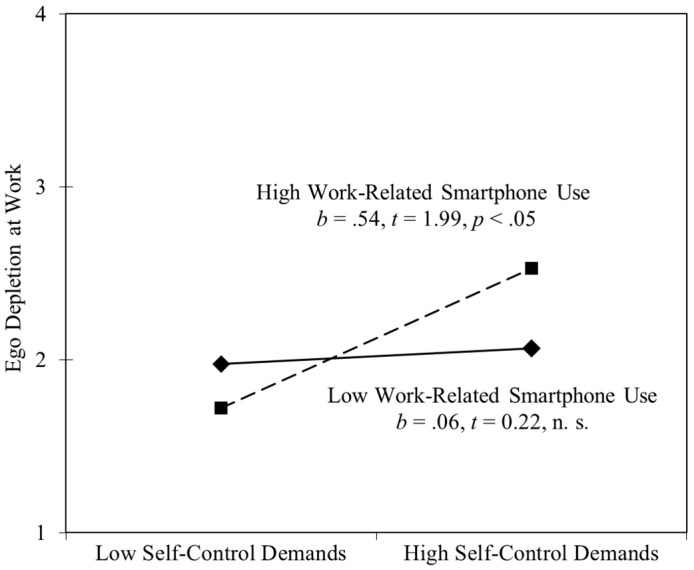
Two-way interaction of work-related smartphone use and next-day self-control demands on next-day ego depletion at work. High and low values were operationalized by one standard deviation above and below the mean.

To examine the interaction in more detail, we depicted it and conducted simple slope tests as recommended by Preacher, Curran, and Bauer [43]. As illustrated in Figure 2, the interaction pattern is consistent with Hypothesis 1. Participants experienced their highest levels of ego depletion when both work-related smartphone use and self-control demands were high. More precisely, when work-related smartphone use was high (+1 SD), self-control demands were positively related to ego depletion. In contrast, when work-related smartphone use was low (−1 SD), this relationship did not reach significance. Thus, as predicted, work-related smartphone use during non-work time strengthened the adverse effects of self-control demands on ego depletion at work the next day.

### 3.2. Testing the Three-Way Interaction between Work-Related Smartphone Use, Self-Control Demands, and Sleep Quality

**Figure 3:**
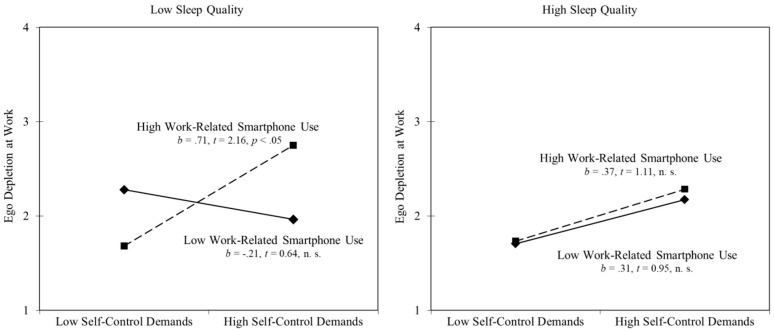
Three-way interaction of work-related smartphone use, sleep quality, and next-day self-control demands on next-day ego depletion at work. High and low values were operationalized by one standard deviation above and below the mean.

Hypothesis 2 predicted that sleep quality moderates the interaction effect of work-related smartphone use and self-control demands. In support of this prediction, the three-way interaction term was significantly related to ego depletion (γ = −0.39, *p* < 0.05; cf. Model 4 in Table 2) and improved model fit (Δ − 2*log(df) = 6.617(3); *p* < 0.10). Figure 3 depicts the pattern of the three-way interaction: Work-related smartphone use at home only strengthened the next-day relationship between self-control demands and ego depletion at work when employees reported low sleep quality (−1 SD). When sleep quality was high (+1 SD), there was no significant relationship between self-control demands and ego depletion at work the next day, regardless of work-related smartphone use. Controlling for working time (full-time versus part-time) did not change the pattern of results.

## 4. Discussion

### 4.1. Summary of the Results

We conducted a daily diary study to examine interaction effects between work-related smartphone use, sleep quality, and self-control demands at work in the prediction of employees’ ego depletion at work. Our findings indicate that work-related smartphone use can affect employees’ self-control processes on the following working day. Specifically, after an evening with high-work-related smartphone use at home, dealing with self-control demands at work can cause disproportionate levels of ego depletion. This finding suggests that work-related smartphone use and self-control demands draw on and deplete the same regulatory resource and supports the notion that resource decrements are transferred between life domains [7]. Moreover, we found that the interaction between work-related smartphone use and self-control demands is moderated by sleep quality: On days when employees experience high sleep quality after having used their smartphones for work intensively, next-day self-control processes at work and associated levels of ego depletion are no longer affected. This evidence is consistent with previous studies demonstrating that sleep ensures returning to work with restored self-control resources on the next day [31,32].

### 4.2. Theoretical Implications

Our research offers some important theoretical implications to the literature on work-related smartphone use, self-control, and sleep. First, the present study advances our knowledge about the mechanism that may underlie the adverse effects of work-related smartphone use. More precisely, the demonstrated interaction effects with self-control demands in the prediction of ego depletion may indicate that work-related smartphone use involves expenditure of self-control resources, and that both demands jointly overtax employees’ limited regulatory resources. We therewith join recent research seeking to identify behaviors and boundary conditions at work that tax regulatory resources (e.g., responding to help requests [44]; time pressure, planning, and decision-making [45]) in order to better understand fluctuations in employees’ well-being.

Second, previous studies on interaction effects between different demands on self-control were foremost conducted on the basis of cross-sectional and longitudinal data with a focus on cumulative effects of chronic demands [8,9]. Since such findings on the between-person level do not automatically indicate the same relations on the within-person level [40], we adopted a daily diary design in our study to further validate existing knowledge. Our findings demonstrate that the strengthening interaction effects also manifest on a daily basis. That is, not only chronic but even short-term (daily) demands on self-control can overtax the limited regulatory resource and cause disproportionate levels of day-specific ego depletion. This strongly underlines the importance of considering day-level processes in employees’ depletion at work.

Third, the current study broadens scholarly knowledge on the consequences of work-related smartphone use by examining spillover effects to the subsequent working day. So far, most prior studies on work-related smartphone use have focused on very proximal effects on impaired psychological well-being in the home domain. To date, there is only one study on work-related smartphone use dealing with spillover effects [6], revealing that work-related smartphone use can impede work engagement on the next day. The current study extends this line of research by demonstrating that work-related smartphone use can affect next-day self-control processes and associated depletion levels at work if individuals fail to replenish their regulatory resources overnight. Our finding strongly suggests that when employees intensively use their smartphone during non-work time at home and experience low sleep quality afterwards, they may not be prepared to effectively respond to demands on self-control on the subsequent working day. Given the close interconnection of work and home in contemporary societies, a deepened understanding of how the demands from one domain can interfere with those from another domain seems essential for both professionals and human resource practitioners.

Finally, we contribute to the growing body of research that reveals the significance of sleep for well-being and organizational behavior (e.g., [6,32,46]). In the context of work-related smartphone use, sleep has mainly been examined as an outcome [47] or as a *mediator* linking smartphone use and strain [6]. The current study, however, provides initial evidence that sleep quality can also serve as a *moderator* mitigating the adverse consequences of work-related smartphone use. By demonstrating that sleep quality can prevent the spillover of drained resources from home to work, and thus offset the adverse effects of work-related smartphone use on self-control processes at work, we further substantiate the role of sleep for replenishing drained regulatory resources [24]. Taken together, the current results suggest that sleep is not only a mediator [6] but also moderator in the link between work-related smartphone use and depletion.

### 4.3. Practical Implications

Apart from theoretical contributions, our research has also some practical implications for employees and organizations. First, findings of this study strongly suggest that work-related smartphone use during non-work time drains limited regulatory resources and thus holds risks for well-being and subsequent self-control functioning. In order to protect themselves against these consequences, employees need to become aware of how long they actually use their smartphone for work during after-hours and, if necessary, reduce the intensity. Apart from that, it seems particularly important that employees engage in behaviors that replenish their regulatory resources, especially on days when work-related smartphone use during non-work time was high. Our study has outlined the benefits of daily sleep quality in alleviating negative effects of work-related smartphone use. To ensure sleep of high quality, scholars suggest taking care of sleep hygiene (e.g., refraining from caffeinated beverages before going to bed, using the bedroom only for sleeping [48]) and developing sleep rituals (i.e., establish fixed times for going to bed and getting up [49]). On days when sleep quality was low, replenishing work breaks on the next day may help to prevent that self-control demands at work become too straining [50].

From an organizational point of view, employers should be cautions when asking employees to respond to work-related issues outside official working time. Since communication technology use at home is strongly influenced by organizational culture [51,52], supervisors should be aware of their position as role models and try to convey adequate expectations on employees’ availability for work during non-work time. A policy, however, that completely disables employees from taking work home (e.g., by shutting down the e-mail server) would impose a restriction to those employees who wish to integrate work and private life. The key may be to ensure that employees understand the risks resulting from work-related smartphone use and utilize it in a way that allows flexibility and work–family integration, but does not pose a threat to their well-being.

Finally, drawing on the finding that employees can return to work with insufficiently recovered regulatory resources, organizations could allocate opportunities for recovery at the workplace (e.g., nap rooms) or encourage employees to take self-initiated short breaks when they are needed [33] in order to ensure effective self-control functioning.

### 4.4. Limitations and Suggestions for Future Research

Despite several contributions, our study is not without limitations. First, because all study variables were operationalized by means of self-report, there is an increased risk that common method variance may have contaminated the observed relations [53]. However, as a high common method variance reduces the probability of detecting interaction effects, the effects of the current study can claim to reflect valid relations rather than common method artifacts [54]. Nonetheless, future research could benefit from using objective reports (e.g., by supervisors or colleagues) or event-sampling (i.e., reporting particular events at work requiring self-control) as alternative measurement approaches of self-control demands. Moreover, future studies could use different operationalizations for work-related smartphone use, such as quantitative measures (duration, frequency, and timing of smartphone use measured via applications), and integrate other characteristics of sleep (e.g., sleep duration) to enhance the validity of the proposed relations.

Second, we did not measure overall time dedicated to work in the evening beyond work-related smartphone use in our study. It is conceivable that participants also spend time on other work-related activities than smartphone use, such as laptops or tablets, which may have additional effects on ego depletion.

Third, based on our finding that work-related smartphone use can trigger self-control processes, we encourage scholars to examine whether psychological resources that have been demonstrated to buffer the adverse effects of self-control demands (e.g., self-control capacity [55]; affective commitment [5]) can also alleviate the adverse effects of work-related smartphone use, and thus protect employees’ well-being. Another interesting goal for future studies could be to examine whether self-control demands at work could also impact work-related smartphone use during the evening. Since some people may need self-control efforts in order to resist impulses to check work-related communications (e.g., e-mails), decreased regulatory resources due to preceding self-control demands at work may make it more difficult for them to refrain from using their smartphone for work-related purposes in the evening.

## 5. Conclusions

Technological advances in recent years have significantly changed the way employees interact with their job. Smartphones are one key facilitator of an instant access to work-related information, making work-related smartphone use during non-work time an emerging issue for employees and organizations. The present study provides new insights into the adverse effects of work-related smartphone use during non-work time. Whereas previous empirical research has mainly focused on same-day consequences, our results suggest that work-related smartphone use during non-work time can affect next-day self-control processes and associated depletion at work. Overall, our study’s findings may offer important impulses for current political debates on work-related use of technology and encourage organizations to more thoroughly consider the complex dynamics between the work and home domains in order to learn how these dynamics can shape employees’ well-being.

## Figures and Tables

**Table 1:** Means, standard deviations, internal consistencies (Cronbach’s Alpha), and intercorrelations of study variables.

Variable		1	2	3	4	5	6
1	Age	–					
2	Gender ^a^	0.04	–				
3	Work-related smartphone use (at home)	−0.01	0.03	*(0.84)*	−0.02	**0.14**	**0.11**
4	Sleep quality	0.08	0.04	−0.01	–	−0.05	**−0.21**
5	Self-control demands (next day at work)	−0.09	−0.05	0.19	−0.01	*(0.93)*	**0.47**
6	Ego depletion (next day at work)	**−0.39**	**−0.31**	0.11	−0.23	**0.52**	*(0.93)*
	M	39.49	1.43	1.51	2.29	2.66	1.80
	SD	13.46	0.50	0.75	0.79	0.76	0.54

Cronbach’s alpha for day-level variables represent the mean internal consistencies averaged over all measurement days. Correlations below the diagonal are person-level correlations (N*_between_* = 63). Correlations above the diagonal are day-level correlations (N*_within_* = 603). Numbers in bold *p* < 0.05. ^a^ Gender (1 = female, 2 = male).

**Table 2:** Multilevel estimates for predicting next-day ego depletion at work.

	Ego Depletion									
	Null Model		Model 1		Model 2		Model 3		Model 4	
Parameter	Estimate	(SE)	Estimate	(SE)	Estimate	(SE)	Estimate	(SE)	Estimate	(SE)
Intercept	1.775 **	0.067	2.077 **	0.186	2.075 **	0.185	2.072 **	0.185	2.070 **	0.186
Age			−0.017 **	0.005	−0.017 **	0.005	−0.017 **	0.005	−0.017 **	0.005
Gender			−0.215 ^+^	0.122	−0.213 ^+^	0.122	−0.213 ^+^	0.122	−0.210 ^+^	0.122
Smartphone use (SU)					0.088 *	0.041	0.069	0.041	0.055	0.040
Sleep Quality (SQ)					−0.121 **	0.038	−0.126 **	0.037	−0.129 **	0.037
Next-day self-control demands (SCDs)					0.290 **	0.066	0.299 **	0.065	0.295 **	0.065
SCDs × SU							0.318 **	0.123	0.325 **	0.124
SCDs × SQ									0.059	0.078
SU × SQ									−0.011	0.064
SCDs × SU × SQ									−0.386 **	0.171
−2*log (lh)	1070.611		1055.378		935.070		928.362		921.745	
Δ − 2*log (lh)			15.233 **		120.308 **		6.708 *		6.617 ^+^	
df			2		3		1		3	

Age and gender are person-level (Level 2) variables; smartphone use, sleep quality, and self-control demands are day-level (Level 1) variables. ^+^ *p* < 0.10. * *p* < 0.05. ** *p* < 0.01. N_within_ = 603. *SE = standard error*.
